# Behavioral problems in psychotic, clinically high-risk, and non-psychotic adolescent patients

**DOI:** 10.1186/s12991-022-00422-1

**Published:** 2022-11-11

**Authors:** Chiara Rogantini, Livio Provenzi, Renato Borgatti, Martina Mensi, Clinical High Risk State For Psychosis Research Group, Melanie Iorio, Marika Orlandi, Arianna Vecchio

**Affiliations:** 1 Department of Brain and Behavioral Sciences, University of Pavia, Pavia, Italy; 2 Developmental Psychobiology Unit, IRCCS Mondino Foundation, Pavia, Italy; 3 Pediatric Neuroscience Center, IRCCS Mondino Foundation, Pavia, Italy; 4 Developmental Psychopathology, IRCCS Mondino Foundation, Pavia, Italy

**Keywords:** Adolescents, Psychosis, Behavioral problems, Youth Self-Report

## Abstract

**Background:**

A growing body of research provides evidence for social and behavioral problems observed among adolescents with psychosis and also as precursors of vulnerability to psychosis, long before the illness onset, especially in females patients. As such, the main aim of the current study was to investigate from a patient perspective the presence of differences in the behavioral problems self-disclosed by psychotic, clinically high-risk, and non-psychotic adolescents. Moreover, since adolescent girls may present higher risk of internalizing problems, we explored the additional role of sex in interaction with psychotic risk or clinical condition in altering the self-disclosed severity of behavioral problems among the three groups of adolescents.

**Methods:**

One-hundred and fifty-eight adolescent patients were interviewed by a trained child neuropsychiatrist applying the Comprehensive Assessment of At Risk Mental States in order to identify a quantitative index of risk for full-blown and attenuated psychosis. All patients self-reported on their behavioral problems filing in the well-validated Italian version of the Youth Self-Report, which quantifies internalizing, externalizing, and total behavioral problems.

**Results:**

Regarding Youth Self-Report’s scores, non-psychotic adolescents had reported lower total and internalizing scores compared to clinically high-risk and psychotic counterparts. Additionally, in our sample a significant group × sex interaction effect emerged for total and internalizing scores: females reported greater risk of total and internalizing behavioral problems, only in the clinically high-risk group.

**Conclusions:**

Higher variability should be expected in the behavioral profile of high-risk adolescents in comparison to psychotic ones. Elevations of internalizing behavioral symptoms, thus, might be considered as a much more relevant risk factor in girls during adolescence.

## Background

It is widely known that adolescence is characterized by a rapid increase in risk for psychosis onset [1, 2] and it is likely to be a crucial period for early intervention and prevention through the identification of the clinically high-risk adolescent population [3]. A growing body of research provides evidence for social and behavioral problems observed among adolescents with psychosis and also as precursors of vulnerability to psychosis, long before the illness onset, especially in female patients [4, 5]. At the same time, behavioral problems may be early rumblings of other psychiatric disorders, too [6]; as such, isolating specific behavioral problems that may be associated with psychotic risk is crucial to improve screening and prevention strategies.

In order to assess the presence of emotional and behavioral problems in adolescents with psychiatric illness a multi-informant approach is recommended as it can provide a multidimensional view of to help clinicians in decision-making and treatment planning [7, 8]. There is a substantial amount of literature that provided multi-informant assessment of behavioral problems in healthy [9] and at-risk adolescents [10, 11]. Previous studies provided multi-informant assessment of emotional and behavioral problems in clinical high-risk adolescents by means of parent-reported instruments, highlighting that internalizing problems are among the most frequent comorbidities [5]. However, the adolescents’ perception of their own emotional and behavioral problems and how the perception diverges among psychotic and non-psychotic patients remains greatly unexplored. Notably, the exploration of these aspects is warranted to increase our comprehension of comorbid emotional and behavioral problems in adolescents with psychotic symptoms and may provide clinicians with a more accurate and complete framework to interpret them in clinical practice and to address a specific individualized treatment. A systematic comparison of behavioral problems among adolescents with psychosis, clinically high-risk, and non-psychotic psychiatric diagnoses is warranted to help clinicians in disentangling specific self-reported behavioral precursors of psychotic risk.

As such, the main aim of the current study was to investigate the presence of statistically significant differences in the behavioral problems self-disclosed by psychotic, clinically high-risk, and non-psychotic adolescents (Aim 1). Moreover, since adolescent girls may present higher risk of internalizing problems [4], we explored the additional role of sex in interaction with psychotic risk or clinical condition in altering the self-disclosed severity of behavioral problems among the three groups of adolescents (Aim 2).

## Methods

### Participants and procedures

A cohort of 158 adolescent patients were consecutively enrolled between December 2017 and August 2021 at the Child Neurology and Psychiatry Unit of the IRCCS Mondino Foundation, Pavia, Italy. The access of the patients to the hospital unit could be for hospitalization, day hospital and/or outpatient visit. Subjects were considered eligible to the study if they presented a diagnosis of psychosis or other mental disorder according to the Diagnostic Statistical Manual (DSM-5) [12] and aged 12 to 18 years. Adolescents were excluded from the study if they presented at least one of the following criteria: intellectual disability, neurological disorders, brain injuries or other medical conditions associated with psychiatric symptoms.

After providing written informed consent, the patients were interviewed by a trained child neuropsychiatrist applying the Comprehensive Assessment of At Risk Mental States (CAARMS) [13], a semi-structured interview that provides validated cut-off to identify adolescents with a quantitative index of risk for full-blown and attenuated psychosis in psychiatric patients. The items assess several psychopathological and functional features clustered into 7 subscales: positive symptoms, cognitive alterations, emotional disturbances, negative symptoms, behavioral changes, somatic-motor changes, general psychopathology. Each subscale is rated on a 7-point Likert severity scale that ranges from 0 (absence of symptoms) to 6 (daily present or high-intensity symptoms) and a 7-point Likert scale for frequency. Patients were assigned to the clinically high-risk group if they presented severe symptoms with moderate frequency, or symptoms of moderate severity but very frequent. For the purposes of the present study, the sample was split into three groups according to CAARMS score: psychotic (*N* = 24, 15%), clinically high-risk (*N* = 63, 40%), and non-psychotic controls (*N* = 71, 45%).

Adolescents self-reported on their behavioral problems filing in the well-validated Italian version of the Youth Self-Report (YSR) [14], which is the most widely used instruments to measure adolescent behavioral problems. These scales have excellent psychometric properties, strong comprehensive reliability and validity evidence [14]. Eight subscales describing specific behavioral problems (see Table 1) are collapsed into three domains: internalizing (INT), externalizing (EXT) and total (TOT) behavioral problems. Each statement is rated on a Likert scale as follows: 0 (not true), 1 (somewhat or sometimes true), or 2 (very true or often true). The continuous T scores can be categorized as clinical risk (above 64 for domains and above 70 for subscale), borderline risk (between 60 and 64 for domains and between 65 and 70 for subscales) and no risk (below 60 for domains and below 65 for subscale).

**Table 1:** Description of the YSR subscales

YSR subscales	Acronym	Items	Description
Anxious/depressed	A/D	13	Feelings of being guilty, nervous, unloved, worthless; it comprises suicidal thoughts
Withdrawal	WD	8	Feelings of being shy, lacking energy, sad, withdrawn
Somatic complaints	SC	11	Feelings of being constipated, dizzy, tired; it comprises aches, headaches, nausea, vomits, stomach-aches, eye and skin problems
Social problems	SP	11	Feelings of being lonely, dependent, not getting along, jealous, teased, not liked, clumsy
Thought problems	TP	15	It comprises hallucinations, delusions, repetitive behavior or strange behavior, feelings of mind off, sleep problems
Attention problems	AP	10	It comprises troubles in concentrating and paying attention, filing to finish, being impulsive and day dreaming, confused
Rule-breaking behaviors	RB	17	It comprises drug and alcohol abuse, rule-breaking, lies and cheats, lacking guilt, having bad friends, running away, setting fires, stealing, vandalism
Aggressive behaviors	AB	18	It comprises disobeying, verbal and somatic fighting, arguing, being suspicious, mean, stubborn, mood changing

*YSR* Youth Self-Report [14]

### Analytical plan

#### Aim 1: group effect

A univariate analysis of variance (ANOVA) was used to assess the presence of statistically significant differences in the self-disclosed TOT score among psychotic, risk, and control adolescents. The presence of a significant effect was further explored with independent-sample *t*-test comparisons, applying Bonferroni correction. Furthermore, two separate ANOVAs were used to assess the presence of statistically significant differences in the self-disclosed INT and EXT scores among psychotic, risk, and control adolescents. Fisher’s *F* coefficient was used to test mean difference and *n*^2^_*p*_ to estimate the dimension of the effect. Significant effects was further qualified with independent-sample *t*-test comparisons, applying Bonferroni correction. Simple effects were estimated with Cohen’s *d*.

#### Aim 2: group × sex effect

Separate ANOVAs with two factors (group, 3 levels: psychotic, risk, controls; sex, 2 levels: males, females) were used to assess the interactive effect of group and sex on TOT, INT, and EXT scores. Fisher’s *F* coefficient was used to test mean difference and *n*^2^_*p*_ to estimate the dimension of the effect. Significant effects was further qualified with independent-sample *t*-test comparisons, applying Bonferroni correction. Simple effects were estimated with Cohen’s *d*.

All the statistical analyses were conducted using Jamovi 2.2.5.0 for Windows [15] (The Jamovi Project, 2021), setting *p* < 0.05.

## Results

### Sample description

**Fig. 1:**
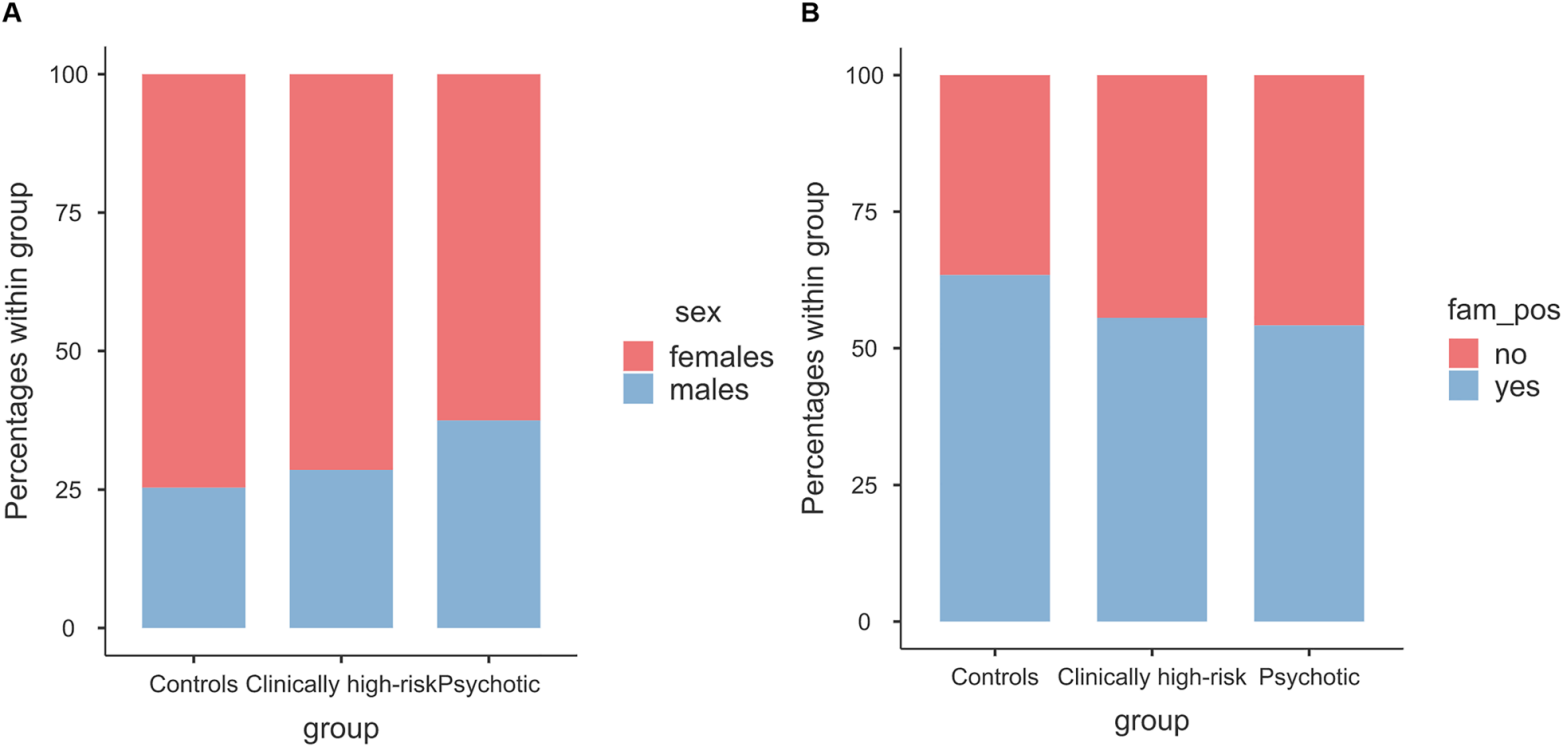
Distribution of sex (**A**) and familiarity for psychiatric disorders (**B**) by group

The sample included 158 subjects (113 females, 72%; mean age 15.22 [range: 12.0–17.9]). The majority of subjects were Caucasians (98.1%). Familiarity for psychiatric diseases was reported in the 59% of the sample (*n* = 93). Table 2 reports comorbidities by group with Chi-square comparisons. Groups did not differ for sex distribution, *Χ*^2^ = 1.30, *p* = 0.522 (see Fig. 1A), and familiarity for psychiatric diseases, *Χ*^2^ = 1.10, *p* = 0.577 (see Fig. 1B).

**Table 2:** Comorbidities split by group

	Controls *N* = 71		Clinically high-risk *N* = 63		Psychotic *N* = 24		Comparison	
	*N*	%	*N*	%	*N*	%	*X*^2^	*p*
Neurodevelopmental disorders	6	8.5	5	7.9	4	16.7	1.70	0.426
Bipolar disorder	2	2.8	6	9.5	2	8.3	2.72	0.256
Depressive disorders	23	32.4	35	55.6	4	16.7	13.60	0.001
Anxiety disorders	22	31.0	19	30.2	2	8.3	5.10	0.078
Obsessive–compulsive disorder	4	5.6	7	11.1	1	4.2	1.90	0.387
Post-traumatic disorder	2	2.8	1	1.6	0	0.0	0.82	0.664
Dissociative disorder	2	2.8	6	9.5	1	4.2	2.92	0.232
Eating disorders	16	22.5	19	30.2	3	12.5	3.13	0.209
Conduct disorder	4	5.6	2	3.2	1	4.2	0.48	0.786
Substance use disorder	1	1.4	3	4.8	0	0.0	2.26	0.324
Personality disorders	12	16.9	15	23.8	1	4.2	4.66	0.097

### Aim 1: group effect

**Fig. 2:**
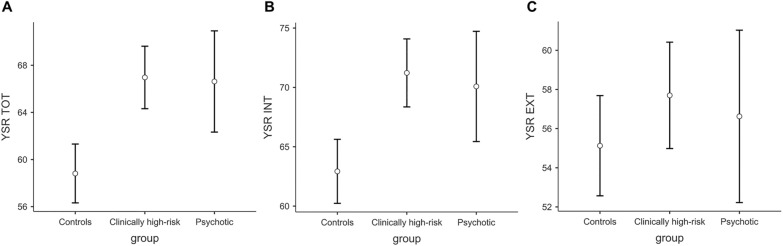
Self-reported YSR scores by group. *YSR* Youth Self-Report [14], *TOT* total behavioral problems, *INT* internalizing behavioral problems, *EXT* externalizing behavioral problems

Table 3 reports the descriptive statistics for YSR scores by group. A significant difference among groups emerged for YSR TOT, *F*(2,155) = 11.20, *p* < 0.001, *n*^2^_*p*_ = 0.13. The *t*-test comparisons revealed that controls had reported lower YSR TOT scores compared to clinically high-risk, *t*(155) = − 4.42, *p* < 0.001, *d* = -0.76, and psychotic counterparts, *t*(155) = − 3.10, *p* = 0.007, *d* = − 0.73 (see Fig. 2A). No significant difference emerge between clinically high-risk and psychotic adolescents.

**Table 3:** Descriptive statistics for the YSR scores by group

	Controls *N* = 71				Clinically high-risk *N* = 63				Psychotic *N* = 24			
	Mean	SD	Min	Max	Mean	SD	Min	Max	Mean	SD	Min	Max
YSR TOT	58.8	11	32	83	67	10.5	43	92	66.6	10	36	87
YSR INT	62.9	11.8	36	90	71.2	11.1	46	91	70.1	11.6	42	86
YSR EXT	55.1	10.5	34	80	57.7	11.5	34	93	56.6	10.5	37	77

*YSR* Youth Self-Report [14], *TOT* total behavioral problems, *INT* internalizing behavioral problems, *EXT* externalizing behavioral problems

Groups also differed for YSR INT, *F*(2,155) = 9.48, *p* < 0.001, *n*^2^_*p*_ = 0.11. The *t*-test comparisons revealed that controls had lower YSR TOT scores than clinically high-risk, *t*(155) = -4.16, *p* < 0.001, *d* = − 0.72, and psychotic individuals, *t*(155) = − 2.63, *p* < 0.028, *d* = − 0.62 (see Fig. 2B). No significant difference emerged between clinically high-risk and psychotic adolescents.

No statistically significant differences emerged for YSR EXT (see Fig. 2C).

### Aim 2: group × sex effect

**Fig. 3:**
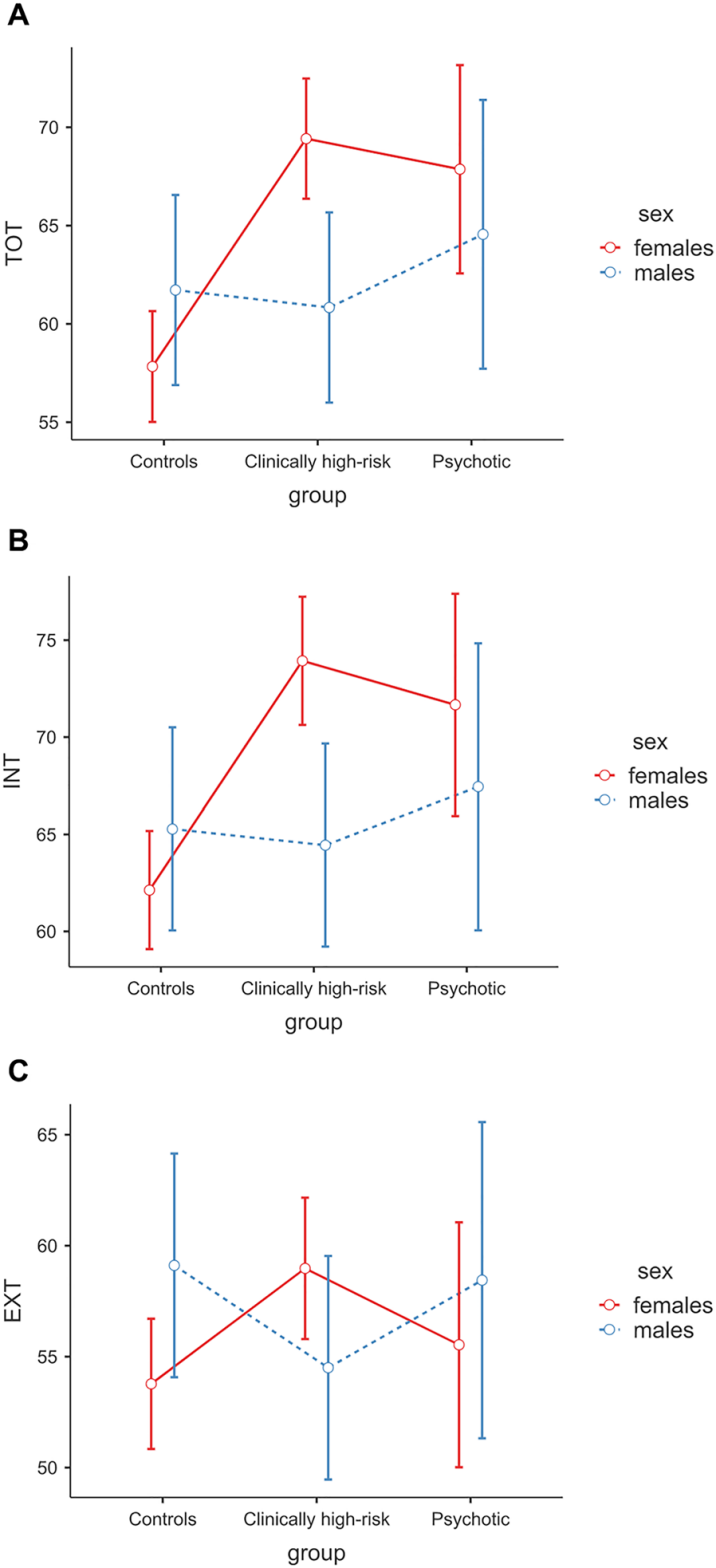
Self-reported YSR scores by group and sex. *YSR* Youth Self-Report [14], *TOT* total behavioral problems, *INT* internalizing behavioral problems, *EXT* externalizing behavioral problems

A significant group × sex interaction effect emerged for YSR TOT, *F*(2,152) = 4.77, *p* = 0.010, *n*^2^_*p*_ = 0.06. Post hoc comparisons revealed the presence of a tendency to statistically significance between males and females only for the clinically high-risk group, *t*(152) = 2.97, *p* = 0.053, *d* = 0.83 (see Fig. 3A). Similarly, a significant group × sex interaction effect also emerged for YSR INT, *F*(2,152) = 4.19, *p* = 0.017, *n*^2^_*p*_ = 0.05. Post hoc comparisons revealed the presence of a significant difference between males and females only for the clinically high-risk group, *t*(152) = 3.03, *p* = 0.043, *d* = 0.85 (see Fig. 3B). No significant interaction effect emerged for YSR EXT (see Fig. 3C).

## Discussion

The present study investigated the presence of significant differences in the self-reported behavioral problems of psychotic adolescents, clinically high-risk peers, and non-psychotic controls. The main findings suggest that (1) psychotic and clinically high-risk adolescents might show similar levels of total and internalizing behavioral problems; (2) non-psychotic controls exhibit lower levels of total and internalizing behavioral problems compared to psychotic and clinically high-risk counterparts, and that (3) the three groups might not differ in externalizing symptoms. In line with previous reports [4], this finding advises that the presence of elevated internalizing symptoms should not be underestimated by clinicians as it might be a primary signals of risk for a psychotic disorders, in presence of other well-known risk factors. Moreover, the onset of a psychotic disorder among adolescents is frequently manifested by negative psychotic symptoms (e.g., abulia, alogia, anhedonia) [16] that are mistaken for depressive symptoms and therefore could lead to a diagnostic delay. It is therefore crucial that clinicians dealing with adolescent psychiatry recall that even clinically high-risk adolescents may express a high presence of internalizing symptoms, which could embody prodromal symptoms of a following psychotic breakdown.

Additionally, we also showed that (4) total and internalizing behavioral problems might be especially elevated in clinically high-risk girls as compared to boys from the same clinical group. This finding suggests that higher variability should be expected in the behavioral profile of high-risk female adolescents in comparison to psychotic ones. Elevations of internalizing behavioral symptoms, thus, might be considered as a much more relevant risk factor in girls during adolescence, altering the self-disclosed severity of behavioral problems among this group of patients. Conversely, when psychotic symptoms are absent or stabilized in a syndromic condition, sex differences appear to be less critical in differentiating the behavioral problems profile of boys and girls.

This study provides some limitations. Firstly, the use of self-reported measure of behavioral problems may have made the assessment of the same hardly unbiased; this concern may limit our ability to generalize about the results and highlights the need to implement the diagnostic evaluation by adding assessments performed by the clinician. Secondly, the sample size is imbalanced, being skewed towards girls. Nonetheless, this issue is at least partially controlled by our analyses, since we have tested for the presence of imbalanced distribution of patients’ sex among the CAARMS-defined groups and there was no significant difference.

## Conclusions

These findings, although preliminary and obtained in a small sample of Italian adolescents, contribute to highlight the importance of considering behavioral problems as potential critical factors in the early detection of psychotic transition in pediatric patients. Since the onset of a psychotic disorder in adolescence is often deceptive and characterized by negative symptoms, externalizing symptoms appear to be less effective in disentangling among adolescents who present a diagnosis of psychosis, those who are at risk, and those how have other psychiatric conditions. As clinicians become more aware of the signaling role of adolescents’ behavioral problems they may be more able to screen, identify, and treat high-risk patients with timely and preventive interventions.

## Data Availability

The datasets generated and analyzed during the current study are available in the ZENODO repository: 10.5281/zenodo.5902821.

## Abbreviations

DSM-5: Diagnostic Statistical Manual
CAARMS: Comprehensive Assessment of At Risk Mental States
YSR: Youth Self-Report
TOT: Total behavioral problems
INT: Internalizing behavioral problems
EXT: Externalizing behavioral problems
